# Interorganellar Membrane Microdomains: Dynamic Platforms in the Control of Calcium Signaling and Apoptosis

**DOI:** 10.3390/cells2030574

**Published:** 2013-08-02

**Authors:** Ida Annunziata, Alessandra d’Azzo

**Affiliations:** Department of Genetics and Tumor Cell Biology, St. Jude Children’s Research Hospital, Memphis, TN 38105, USA

**Keywords:** interorganellar membrane microdomains, MAMs, ER-PM junctions, GM1-ganglioside, Ca^2+^ signaling, apoptosis

## Abstract

The dynamic interplay among intracellular organelles occurs at specific membrane tethering sites, where two organellar membranes come in close apposition but do not fuse. Such membrane microdomains allow for rapid and efficient interorganelle communication that contributes to the maintenance of cell physiology. Pathological conditions that interfere with the proper composition, number, and physical vicinity of the apposing membranes initiate a cascade of events resulting in cell death. Membrane contact sites have now been identified that tether the extensive network of the endoplasmic reticulum (ER) membranes with the mitochondria, the plasma membrane (PM), the Golgi and the endosomes/lysosomes. Thus far, the most extensively studied are the MAMs, or mitochondria associated ER membranes, and the ER-PM junctions that share functional properties and crosstalk to one another. Specific molecular components that define these microdomains have been shown to promote the interaction in trans between these intracellular compartments and the transfer or exchange of Ca^2+^ ions, lipids, and metabolic signaling molecules that determine the fate of the cell.

## 1. Introduction

A characteristic aspect of the eukaryotic cell is the complex network of intracellular compartments or organelles with distinct biological functions and delimited by a lipid bilayer that insulates their contents from the cytoplasm. Although seemingly static in appearance on electron micrographs and acting independently from one another, organelles are in a state of constant remodeling and communication, which is often achieved at tethering sites between their individual membranes, where two engaged organelles become closely juxtaposed without undergoing fusion [1,2]. It is now well documented that these membrane contact sites are recognizable and active in many organisms and cell types [3,4,5,6]. They define dynamic platforms for the synthesis and redistribution of lipids, signaling molecules, and ions (e.g., calcium, Ca^2+^), when rapid exchange between organelles is required without the involvement of vesicle trafficking. Although still little is known about their lipid and protein compositions and refined mode of action, the best studied membrane contact sites are those that are formed between the vast membrane network of the endoplasmic reticulum (ER) and the membranes of other cellular compartments, particularly the plasma membrane (PM) and the mitochondria. Common functions of these membrane contact sites are the regulation of Ca^2+^ dynamics that assures the homeostasis of intracellular Ca^2+^ concentration, and the synthesis/shuttling of lipids and lipid intermediates, i.e., phospholipids and (glyco)sphingolipids, such as ceramide and GM1-ganglioside (GM1). There is now evidence that these functions of membrane contact sites can be interrelated, given that changes in lipid content and concentration may alter the activity of Ca^2+^ binding proteins and Ca^2+^ channels/pumps. Cellular Ca^2+^ signals are initiated primarily with a rapid increase in cytosolic Ca^2+^ concentration that in turn controls downstream cellular processes, such as gene transcription, cell proliferation and metabolism, and neural transmission [7,8,9]. Disturbance of Ca^2+^ signaling, specifically at membrane contact sites, may put the cell in a state of stress and ultimately lead to cell death. 

In this review, we give a condensed overview of the structural and molecular aspects of interorganellar membrane microdomains, focusing primarily on those formed between the ER and the mitochondria, as major Ca^2+^ store compartments, and on their cross talk with the PM. We will discuss how their membrane topology and composition, especially in relation to the amount of GM1-ganglioside, affects Ca^2+^ signaling and in turn controls cell fate.

## 2. ER-PM Junctions

Keith Porter and George Palade first identified these junctions [10] in electron micrographs of striated muscle cells, where Ca^2+^ flux at these two juxtaposed membrane compartments is crucial for excitation-contraction coupling. Initially thought to be present exclusively in such specialized cells like muscle cells, ER-PM junctions, also known as PAMs for PM associated ER membranes [6], have later been recognized morphologically in a variety of organisms and cell types from yeast to mammals [1,11,12,13,14], and the quest for the identification of their molecular components began. One of the early-characterized junctional complexes is the one formed by the ER-resident STIM (stromal interaction molecule) proteins and the PM-resident Ca^2+^ channel Orai1 or CRAC (Ca^2+^ release activated Ca^2+^ channel) in the store operated Ca^2+^ entry or SOCE pathway (reviewed in [1]). Following Ca^2+^ depletion of ER stores, STIM1 acts as the Ca^2+^ sensor at the ER membrane and accumulates into punctae close to the PM. Upon translocation to the PM, STIM1 promotes Ca^2+^ influx interacting with Orai1. This specific protein-protein tethering allows for the rapid transduction of signaling and also for the refilling of the ER Ca^2+^ pool without affecting cytosolic Ca^2+^ concentration. 

In cardiac and skeletal muscle cells analogous sarcoplasmic reticulum (SR)-PM junctions have been implicated in the process that allows the transmission of signals from the PM to the SR and the local release of Ca^2+^ from the SR Ca^2+^ stores. Several proteins have been described as components of these SR-PM junctions, although those that are believed to play a role in tethering these junctions are the junctophilins. The latter comprise a family of ER-localized integral membrane proteins containing several MORN (membrane occupation and recognition nexus) motifs that appear to bind phosphoinositide lipids at the PM side [15]. Furthermore, in yeast, large regions of the PM have also been observed in close proximity to the cortical ER [11]. Using quantitative proteomics analyses Manford* et al.* [14] have recently described several proteins, which tether the PM to the cortical ER, regulating cell signaling and ER structure. This biochemical screening identified the VAP (vesicle-associated membrane protein-associated protein) orthologs Scs2 and Scs22 on the PM side, and the yeast proteins Ist2, the tricalbin proteins Tcb1 and Tcb3 on the ER side. Scs2 and Sc22 have already been implicated in ER-PM tethering [16] and contain a sequence motif, which binds to a domain present in lipid transfer proteins. Ist2 and the tricalbins have both transmembrane domains and cytoplasmic lipid-binding domains, and physically interact with Scs2 and Scs22, hence defining the ER-PM tethering sites. Deletions of all these genes in yeast abolish the connection between the ER and the PM with considerable reduction of cortical ER and its redistribution into internal structures [14]. In addition, loss of ER-PM contacts causes a dramatic accumulation of phosphatidylinositol 4-phosphate at the PM and activation of the UPR (unfolded protein response), indicating that maintaining these junctions is critical for cell and organelle functions. 

## 3. Mitochondria Associated ER Membranes or MAMs

The most extensively characterized interorganellar membrane contact sites from a structural, biochemical and functional standpoint are those at the interface between the ER and the mitochondria, defined as MAMs or mitochondria associated ER membranes [17]. These microdomains represent highly dynamic signaling and metabolic platforms that are implicated in fundamental cellular processes, such as lipid biosynthesis and transport, Ca^2+^ signaling, energy metabolism, cell survival and apoptosis. Structural and functional analogies between the MAMs and the ER-PM junctions suggest a strict interplay between these cellular compartments, especially as it pertains to Ca^2+^ dynamics and lipid shuttling.

### 3.1. Lipid Biosynthesis and Transport at the MAMs

Morphological evidence for the physical juxtaposition between ER and mitochondria emerged already in the 1960s [18] but it was experimentally proven in the early 1990s when it was shown that fractions of the ER co-purified with mitochondria in velocity sedimentation assays [19]. These authors were the first to show that these co-sedimenting fractions were enriched in enzymes responsible for the synthesis of lipids, a finding that proposed the MAMs as sites of non-vesicular lipid transfer between the ER and the mitochondria. One of the enzymes found to be enriched in these microdomains is the phosphatidyl-serine synthase, which localizes at the ER face of the MAMs, opposite to phosphotidyl-serine decarboxylase at the mitochondrial side. This strategic positioning of the two enzymes enables the regulated transport of phosphatidyl-serine from the MAMs to the mitochondria, a rate-limiting step for the synthesis of phosphatidyl-ethanolamine within the mitochondria. It also ensures the controlled supply of phospholipids to the mitochondria, which is necessary for maintaining mitochondrial membrane integrity following fusion and fission of these organelles [20,21,22].

The MAMs are also enriched in enzymes necessary for cholesterol and steroid biosynthesis [19], as well as ceramide production [20,23]. Although not yet proven, specific lipid carriers may orchestrate trafficking of lipids and their intermediates between ER and mitochondria. Alternatively, physical flipping of the lipids may occur between the juxtaposed membranes if they are in sufficiently close proximity. Lipids may also transfer through macromolecular complexes present at the MAMs [21]. The latter mechanism has not been found yet in mammalian cells but has been reported in yeast. A macromolecular complex was recently identified through a synthetic screening for yeast mutants that do not grow unless they express a synthetic fusion protein that artificially tether ER and mitochondrial membranes at contact sites. This complex, named ERMES (ER-mitochondria encounter structure), functions not only as a molecular tether between the two organelles, but also for the shuttling of phospholipids [24,25]. Cells missing individual components of the ERMES complex show reduced levels of mitochondrial phosphatidyl-ethanolamine and cardiolipin, demonstrating that all the components of the complex are functionally related to phospholipid biosynthesis and their transfer [24].

### 3.2. Ca^2+^ Signaling between ER and Mitochondria at the MAMs

ER and mitochondria are the major intracellular Ca^2+^ stores and function synergistically to maintain the cytosolic ion concentration in the nanomolar range for the proper physiology of the cell. As primary site of Ca^2+^ storage, the ER controls Ca^2+^-dependent physiological processes, such as post-translational protein folding, protein maturation and membrane stability [9,26]. On the other hand, mitochondria buffer and transiently store cytosolic Ca^2+^ in a process that is essential for mitochondrial bioenergetic activity [27,28,29,30]. The intimate interplay between the two organelles at the MAMs offers the most efficient means for the transfer of Ca^2+^ from the ER to the mitochondria at the opening of the inositol 1,4,5-triphosphate (IP3)-sensitive Ca^2+^ channel [31,32] This configuration ensures that Ca^2+^ either remains local or that Ca^2+^ waves dissipate from neighboring to distant mitochondria, thereby avoiding mitochondrial Ca^2+^ overload [33,34,35,36]. 

The uptake of Ca^2+^ by the mitochondria occurs at the expenses of an electrical potential across mitochondrial membranes [34,37]. Originally referred to as mitochondrial Ca^2+^ uniporter, the main transporter for mitochondrial Ca^2+^ uptake has now been found to consist of a complex of at least two proteins: MICU1 (mitochondrial calcium uptake 1), which has properties of a Ca^2+^ uniporter or regulates its activity [38], and the transmembrane protein CCDC109A, renamed MCU for mitochondrial calcium uniporter [39,40]. Silencing of MICU1 abolishes Ca^2+^ uptake into the mitochondria, without impairing mitochondria respiration or affecting mitochondria membrane potential. On the other hand, overexpression of MCU increases Ca^2+^ uptake while silencing of the protein reduces mitochondrial Ca^2+^ intake [39,40].

Once in the mitochondria, Ca^2+^ acts as an important cofactor that activates and regulates Ca^2+^-dependent dehydrogenases of the Krebs cycle for maintenance of mitochondrial respiration and ATP production [29,30]. ATP can then stimulate Ca^2+^ receptors on the ER membranes or can be used by the ER Ca^2+^ ATPases to pump Ca^2+^ back into the ER. The discharge of Ca^2+^ into the cytosol, instead, follows an ion gradient and is mediated by the antiporter Letm1 (Leucine zipper-EF-hand containing transmembrane protein 1) [37,41]. As Ca^2+^ levels are linked to different forms of cell death and mitochondria are primary effectors of the intrinsic apoptotic process, it is conceivable that disturbance of the MAMs homeostatic control of Ca^2+^ buffering between ER and mitochondria constitutes a potent molecular activator of apoptosis. 

## 4. Protein Components of the MAMs

Specific ER and mitochondrial proteins co-localize at the MAMs, but the molecular mechanism(s) by which tethering between the two organellar membranes is achieved is not yet fully elucidated. In some instances, it might depend on the interaction of proteins and lipids located at the ER and the mitochondrion sides, which physically connect the membranes of the two organelles [35,42]. For example, the IP3R-1 at the ER face of the MAMs is bridged to VDAC-1 (voltage-dependent anion channel-1) at the OMM (outer mitochondrial membrane) by GRP75 (glucose-regulated-protein 75), a molecular chaperone also present in the mitochondrial matrix [43]. Association of these proteins confers stability to the tethering complex and allows the formation of a pore that favors Ca^2+^ transfer from the ER to the mitochondria [35,43]. 

**Figure 1:**
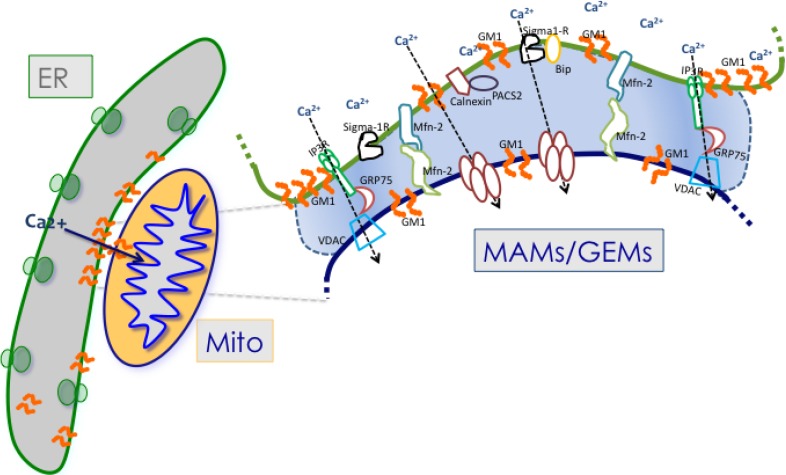
Schematic representation of some of the proteins and lipids localized to the MAMs. Example of proteins localized to the MAMs (such as Mfn2, Sigma 1R, BiP, IP3R1, VDAC, and GRP75) that have been shown to regulate Ca^2+^ signaling and the topology of ER-mitochondria microdomains. GM1 is the only lipid at the MAMs, which influences both Ca^2+^ flux and the number of contact sites.

**Abbreviations**: GM1, GM1-ganglioside; IP3R, inositol 1,4,5- triphosphate receptor; GRP75, glucose related protein 75; VDAC, voltage-dependent anion channel; Sigma-1R, Sigma 1 receptor; Mfn2, mitofusin 2; PACS2, phospofurinic acidic cluster sorting protein-2.

Additional proteins that have been found to localize at the MAMs include the chaperones Sigma-1R (receptor), calnexin, BiP and calreticulin, the trafficking molecule Rab32, the parkinson disease-related protein DJ-1 and the multifunctional sorting protein PACS-2 (phosphofurin acidic cluster sorting protein-2) [44,45,46,47,48] (Figure 1). Recently, also the PML (promyelocytic leukemia) tumor suppressor protein has been shown to have an extranuclear activity that is controlled by its localization in a multi-protein complex at the surface of the ER and at the MAMs [49]. Of these MAM-localized proteins, Sigma-1R functions as regulator of intra- and inter-organellar membrane microdomains, because it can reside at three different membrane sides, the ER, the MAMs, and the ER-PM interface, in response to specific cellular stimuli [45,50]. Under physiological conditions, Sigma-1R is present at the ER face of the MAMs in complex with the chaperone BiP. Upon glucose starvation or activation of the IP3R-3, Sigma-1R dissociates from BiP and stabilizes the IP3R-3 at the MAMs, thereby favoring Ca^2+^ transfer from the ER to the mitochondria [45]. Interestingly, overexpression of Sigma-1R induces its translocation from the MAMs to the PM face of the ER-PM junctions, where it interacts with and modulates the activity of ion channels, receptors and kinases [50,51,52,53,54]. Similarly, interaction of calnexin with PACS-2 regulates the function of the chaperone by promoting its localization to the ER and the MAMs. PACS2 downregulation, instead, causes the relocalization of calnexin to the PM, affecting the levels of this chaperone at the MAMs [46]. Although not yet experimentally proven, diverting calnexin to the PM likely diminishes the amount of the protein available for interaction with the IP3-R, thereby influencing the activity of the channel. Even though neither of these MAM-localized proteins have been shown to have an intrinsic tethering capacity, they can still affect ER-mitochondria contact sites through their dynamic regulation of Ca^2+^ concentration at the MAMs, triggering an apoptotic cascade when deregulated [55]. Furthermore, the fact that the aforementioned proteins under either physiological or pathological conditions can redistribute to both the MAMs and the ER-PM junctions reiterates the strict interplay between these microdomains and their components.

It is noteworthy that Ca^2+^ concentration at the MAMs also controls the physical distance between the ER and mitochondrial membranes [17,35,56]. This occurs via the release of Ca^2+^ from the ER that arrests the movements of neighboring mitochondria, thus easing their close opposition to the ER membrane, which favors mitochondrial Ca^2+^ buffering. 

Going full circle, the distance between the two juxtaposed membranes at the MAMs, in turn, regulates the extent of Ca^2+^ flux. The latter has been demonstrated by the use of synthetic linkers that artificially tethered the two organelles [57]. Decreasing the distance of apposition between ER and mitochondrial membranes leads to mitochondrial Ca^2+^ overload, whereas increasing the distance impairs mitochondrial function resulting in cell death [57]. 

The only ER-mitochondria bona fide tether discovered so far is Mfn2 (mitofusin-2), a dynamin-related protein and potent effector of mitochondrial fusion, which is mutated in the Charcot-Marie-Tooth IIa disease [58]. Mfn2 is located in the OMM, is highly enriched in the MAMs and is also present in the ER, albeit in low amounts [59]. Loss of function or silencing of Mfn2 in MEFs and HeLa cells increases the distance between ER and mitochondria with consequent reduction of Ca^2+^ flux at the MAMs [59]. In agreement with this observation, Mfn2 silencing was shown to disengage ER and mitochondria at the MAMs, thereby reducing mitochondrial Ca^2+^ overload and mitochondria-mediated cell death [35]. Together these results establish Mfn2 as a physical tether between ER and mitochondrial membranes at the MAMs and emphasize the reciprocal connection between the topology of the MAMs and their ability to control Ca^2+^ flux [35,59]. 

## 5. Lipid Components of the MAMs

Although it is assumed that ER and mitochondrial membranes are linked primarily by trypsin-sensitive structures, implying an exclusively protein-based connection, there is now growing evidence for the combined role of proteins and lipids, specifically cholesterol and glycosphingolipids (GSLs), in enabling the formation of the MAMs, hence influencing their physiological function(s) [35,60]. Cholesterol is a central constituent of biological membranes, which regulates a plethora of cellular processes, including gene transcription and signal transduction. It is noteworthy that cholesterol is particularly abundant at the MAMs where it associates with Sigma-1R at cholesterol-enriched subdomains of these microdomains [61]. GSLs have a high melting temperature and in cellular membranes are not combined with glycerophospholipids, but rather tend to cluster and form microdomains [62]. This concept is further supported by the existence of so called “lipid membrane domains”, a dynamic assemblage of sphingolipids, cholesterol and proteins that dissociate and associate rapidly and form functional clusters in cell membranes [63]. These clusters provide highly efficient lipid-protein modules, which operate in membrane trafficking and cell signaling [64]. The recent discovery that accumulation of the GSL GM1 at the ER membranes promotes the juxtaposition of ER and mitochondria at the MAMs, and in turn increases Ca^2+^ transfer between these organelles, underscores the cooperation of lipids and proteins in regulating physiological pathways at membrane contact sites that favor either cell survival or cell death [35]. 

## 6. GM1-Ganglioside and Ca^2+^ Regulation

Gangliosides comprise a class of sialic acid-containing GSLs, which occur ubiquitously in vertebrate cells. They are major constituents of the PM and are also present in the nuclear envelop [9,65,66]. They are particularly abundant in the nervous system where they account for approximately 10–12% of the total lipid contents of neuronal membranes. Structurally, gangliosides are composed of a common hydrophobic ceramide moiety, which serves as membrane anchor, and a hydrophilic oligosaccharide chain, which varies in length and composition and includes one or more sialic acids. Because of their exclusive and asymmetric distribution in neuronal membranes, gangliosides have been shown to play important roles in basic neuronal functions, such as synaptic transmission and neuritogenesis [9,67]. In addition, they have been implicated in the regulation of cell growth, and function as receptors or co-receptors for cytokines, toxins, viruses, and bacteria [68,69,70,71]. 

Among the GSLs, GM1 is the only ganglioside that has been experimentally shown to modulate Ca^2+^ concentration in intracellular stores [9,35,72,73,74]. GM1 resides abundantly in the PM, particularly of neurons, and in less amounts in the nuclear membrane [75,76,77]. Studies in immune cells and neural cells have demonstrated the capacity of GM1 to potentiate the activity of a Na^+^/Ca^2+^ exchanger (NCX) isoform present in the inner nuclear membrane by tightly associating with it [78,79]. These authors proposed that the interaction between NCX and GM1 is favored by the physical proximity of the negatively charged oligosaccharide chain of GM1 and the large loop between the transmembrane regions in NCX, both facing the nucleoplasm [79,80]. This topological configuration is apparently required for the transfer of Ca^2+^ from the nucleoplasm to the nuclear envelope lumen and in turn to the ER, which constitutes an alternative route to that controlled by the ER Ca^2+^ pump SERCA for maintaining Ca^2+^ homeostasis [80,81]. In fact, Jurkat cells lacking nuclear NCX have no or greatly reduced Ca^2+^ transfer from the nucleoplasm to the ER. In addition, cerebellar granule neurons isolated from mice deficient for the GM2/GD2 synthase (GalNAcT^−/−^ mice) that lack complex gangliosides, including GM1, showed elevation of intracellular Ca^2+^ under depolarizing levels of potassium, which is associated with extensive apoptosis [79,82]. Remarkably, the latter phenomenon could be reverted by exogenous administration of GM1, suggesting a cytoprotective function for this ganglioside through its ability to regulate intracellular Ca^2+^ concentration [83].

These, and our own, studies have now established the importance of maintaining physiological levels of GM1 in cell membranes, particularly neuronal membranes, to avoid the initiation of an apoptotic cascade downstream of deregulated Ca^2+^ signaling. 

Increased levels of GM1 above its physiological threshold are as deleterious for neurons as the complete lack of the ganglioside, and activate a cell death pathway dependent on loss of Ca^2+^ homeostasis [35,74]. Pathogenic buildup of GM1 occurs in the lysosomal storage disease GM1-gangliosidosis, a generalized, severe neurosomatic condition caused by deficiency of the GM1-degradative enzyme *β*-gal (*β*-galactosidase) [84]. The disease is associated with widespread neuronal loss and neuroinflammation [74,85,86]. Initial studies in the *β-gal^−/−^* mice have demonstrated that impaired lysosomal degradation of GM1 in neurons is accompanied by the abnormal redistribution of the ganglioside from the PM to ER membranes. Accumulation of GM1 at this site causes depletion of the ER Ca^2+^ store, which effectively activates the UPR, initially as survival mechanism. However, sustained activation of the UPR molecular effectors due to continuous GM1 buildup, and hence release of ER Ca^2+^, ultimately triggers a UPR-mediated apoptotic process [9,74].

## 7. GM1 at MAMs/GEMs and Activation of Cell Death

**Figure 2:**
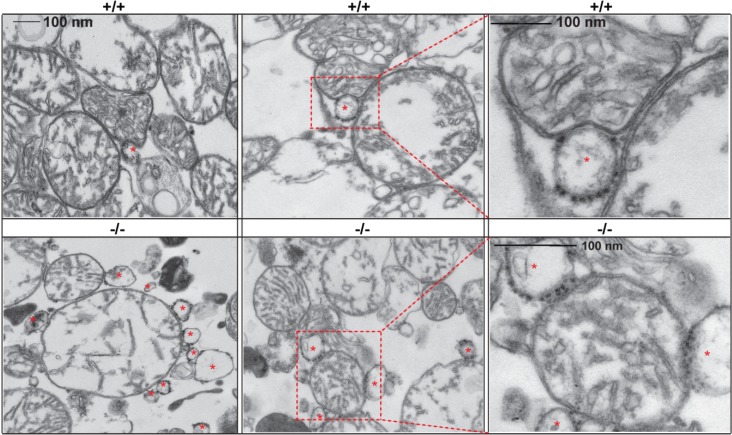
GM1 accumulation favors the apposition between ER and mitochondrial membranes at the MAMs. Representative electron micrographs of crude mitochondria isolated from *β-gal^+/+^* and* β-gal^−/−^* brains. Asterisks mark ER vesicles juxtaposed to mitochondrial membranes.

The finding of the localization of GM1 to the ER membranes prompted a series of studies that eventually identified GM1 as a structural and functional component of the MAMs (Figure 1). Ultrastructural analyses of crude mitochondria isolated from *β-gal^−/−^* mouse brains revealed the presence of an increased number of ER vesicles tethered to mitochondria, compared to the number of this vesicles found associated to wild-type mitochondria (Figure 2). These observations raised the possibility that GM1 accumulation in the ER would potentiate the formation of membrane contact sites between ER and mitochondria, and hence increase their number. Indeed, pure preparations of MAMs isolated from brains of *β-gal^−/−^* mice [35,87] were found to contain high levels of GM1, which is usually undetectable in wild-type preparations [35]. Subsequent extraction of purified MAMs with Triton X-100 to obtain the GEMs (glycosphingolipid enriched microdomains) fraction showed even higher amounts of GM1 in the *β-gal^−/−^* GEMs than in the MAMs, indicating that this ganglioside preferentially segregates in these sub-microdomains. The presence of small amounts of GM1 also in *β-gal^+/+^* GEMs supports the idea that this lipid is a normal constituent of both ER and mitochondrial membranes. 

This research was originally published in *Mol. Cell*. [35].

GEMs are dynamic microdomains present both in the PM and intracellular membranes. They share several protein components with the caveolae and the lipid rafts, and have been proposed to function as signaling platforms by facilitating the clustering of proteins as well as protein–protein interactions [88,89]. This was initially demonstrated in T-cells, where GEMs isolated from crude mitochondria are enriched in another ganglioside, GD3, which cooperates with CD95/Fas in initiating an apoptotic process [90]. Our studies in *β-gal^−/−^* neurons have shown, for the first time, the existence of GEMs within the MAMs and have highlighted a new function of the GEMs as subdomains specific for Ca^2+^ transfer from the ER to the mitochondria mediated by GM1. Similarly to what described for the NCX/GM1 complex, abnormal accumulation of GM1 in the GEMs potentiates the activity of the Ca^2+^ pore composed by the IP3R-1, VDAC-1 and GRP75. The enrichment of the phosphorylated and active form of the IP3R-1 (P-IP3R1) in the *β-gal^−/−^* GEMs, and the co-precipitation of P-IP3R1 with GM1 suggest a physical and functional interaction between these two molecules. Concomitantly, the levels of VDAC-1 and GRP75 were also higher in the *β-gal^−/−^* GEMs than in the wild-type samples, supporting the notion that increased levels of GM1 promote the tethering of the ER-mitochondrial membranes at the GEMs. In these lipid-rich microdomains, high concentration of GM1 likely alters the dynamics of the lipid microenvironment, favoring the segregation or the clustering of an active IP3R-1 along with VDAC1 and GRP75, and promoting the formation of a Ca^2+^ mega-pore. This allows for a more sustained diffusion of Ca^2+^ into the mitochondrial matrix, which disrupts the bioenergetic activities of the organelle, and activates the mitochondrial leg of the intrinsic apoptotic pathway [35]. Mitochondria depolarization, opening of the PTP (permeability transition pore) and MMP (mitochondrial membrane permeabilization), key events in the apoptotic cascade [91,92], have been shown to occur downstream of GM1-dependent Ca^2+^ overload into the mitochondria in *β-gal^−/−^* cells and brain tissue [35]. Opening of the PTP causes the release of apoptogenic factors (like cytochrome *c*), and formation of the apoptosome, which signs the onset of programmed cell death. The fact that GM1-mediated PTP opening and MMP are antagonized by PTP inhibitors and Ca^2+^ chelators, and rescued in cells double deficient for *β-gal^−/−^* and cyclophilin D, a structural component of the PTP, suggest that GM1 acts directly at the pore level in a Ca^2+^-dependent manner [35]. 

The mitochondrial damage that ensues upon abnormal GM1 accumulation in the GEMs also underscores the importance of these microdomains in maintaining a normal electrochemical gradient across mitochondrial membranes. In fact, treatment of *β-gal^−/−^* cells with MBCD (methyl β-cyclodextrin), which efficiently extracts GM1 from the MAMs, rescues opening of the PTP, dissipation of the potential, and apoptosis [35]. A similar outcome can be obtained by silencing Mfn2, which underscores the importance of membrane tethering in eliciting the apoptotic process. Together, these findings reiterate the joined role of both proteins and lipids, particularly GM1, in the regulation of Ca^2+^ flux at the MAMs/GEMs and in Ca^2+^-dependent apoptosis.

## 8. GM1 at the PM and its Relocalization in Intracellular Membranes

It is conceivable that the sequence of events that leads to GM1 buildup at the MAMs/GEMs and ER-mitochondrial apoptosis begins with abnormal levels of GM1 at PM, the site where the ganglioside is primarily localized under physiological conditions. In fact, a question that still remains unanswered is how GM1 redistributes to the ER membranes and hence to the MAMs/GEMs. One hypothesis is that it is transported from the PM to intracellular membranes via caveolae or lipid rafts. Supporting evidence for this is the fact that GM1, as receptor for cholera toxin at the PM, is internalized via caveolar-/non-caveolar- or clathrin-mediated endocytosis and transported to the Golgi compartment and to the ER membrane [93]. A second possibility is that GM1 is directly transferred from the PM to the ER membranes at tethering sites between these two compartments. This would create a continuous supply of GM1 from the ER-PM junctions to the MAMs that could influence the topology of both membranes. Making a parallel with what occurs at the MAMs/GEMs structures in relation to Ca^2+^ regulation in excess of GM1, it is tempting to speculate that an abnormal concentration of GM1 at the ER-PM junctions may also impact on the structure and function of Ca^2+^ channels at these Ca^2+^ microdomains. This could affect homeostatic Ca^2+^ flux between the PM and the cytosol, or the PM and the ER, and hence the physiological refill of ER Ca^2+^ stores.

## 9. Conclusion

It is now widely accepted that intracellular compartments are in constant interaction and communication with one another. This interplay between organelles occurs via membrane tethering sites that come in close vicinity in trans and facilitate the exchange or transfer of molecules and ions to preserve the physiological status of a cell. Membrane microdomains need to acquire or maintain a specific topology and lipid/protein composition in order to be fully active. In contrast, pathological conditions that interfere with their structure and/or function result in cell death. Intriguingly, different organellar membranes may come in close apposition to tether or fuse. We are now beginning to understand that some of the molecular players (*i.e.*, Mfn2) that specify membrane contact sites may regulate both processes in response to certain physiological or pathological cues. Future studies in this area of research promise to uncover new aspects of cell biology that are orchestrated by interorganellar membrane microdomains. 
